# Prevalence and influencing factors of obesity in preschool children in Suzhou, China

**DOI:** 10.23938/ASSN.1105

**Published:** 2025-04-30

**Authors:** Yu Zhang, Xu Zhang, Xiaohua Wang, Zhen Wu, Ying Wang, Hang Ding

**Affiliations:** 1 Suzhou City University School of Urban Governance and Public Affairs Suzhou Jiangsu China; 2 Suzhou City University School of Wellness Industry Suzhou Jiangsu China; 3 Feicui Kindergarten of Suzhou Industrial Park Suzhou Jiangsu China; 4 JinXi Kindergarten of Suzhou Industrial Park Suzhou Jiangsu China; 5 Gongyuanlu Kindergarten of Suzhou Suzhou Jiangsu China

**Keywords:** Preschool Children, Obesity, Risk Factors, Kindergarten, Niños en edad preescolar, Obesidad, Factores de riesgo, Jardín de infancia

## Abstract

**Background:**

The study aims to assess the prevalence of obesity among preschool children in Suzhou, China, and analyze potential risk factors to the condition.

**Methods:**

The study included preschool children aged 3-6 from various kindergartens in Suzhou. Height and weight measurements were used to determine obesity status, while parents completed questionnaires on relevant information. The Chi-square test was applied to compare obesity rates across groups, and stepwise logistic regression analysis identified associated risk factors for obesity.

**Results:**

Eight hundred and forty-eight preschoolers participated. The overall obesity prevalence was 14.0%, with 4.0% categorized as moderately obese (and 7.5% as mildly obese. Boys had a higher obesity prevalence than girls (15.3% vs. 12.7%), and rural children higher than urban children (17.2% vs. 13.1%), although these differences were not statistically significant. Obesity was most prevalent among 3-year-olds (16.1%) and least prevalent among 6-year-olds (11.2%). Logistic regression identified age (boys: OR=0.30; 95%CI: 0.19-0.38, girls: OR=0.24; 95%CI: 0.17-0.36), height (boys: OR=1.21; 95%CI: 1.16-1.26, girls: OR=1.25; 95%CI: 1.18-1.38), and urban location (boys: OR=0.60; 95%CI: 0.34-0.86, girls: OR=0.48; 95%CI: 0.31-0.84) as significant independent predictors for obesity in preschool children in Suzhou, China.

**Conclusions:**

The obesity rate among preschool children in Suzhou is 14.0%. The study highlights lower age, higher height, and rural location as predictor factors of obesity in both sex.

## RESUMEN

**Fundamento:**

El objetivo es evaluar la prevalencia de obesidad entre preescolares de Suzhou (China) y analizar su relación con diferentes factores.

**Métodos:**

En el estudio participaron niños de 3 a 6 años de edad de varios jardines de infancia de Suzhou. Se midieron la altura y el peso para determinar el estado de obesidad, y los padres rellenaron cuestionarios para obtener información relevante sobre estilos de vida. Se compararon las tasas de obesidad mediante Chi-cuadrado y el análisis de regresión logística por pasos identificó los factores de riesgo asociados.

**Resultados:**

Participaron 848 preescolares. La prevalencia global de obesidad fue del 14,0%: 4,0% de moderada y 7,5% leve. La prevalencia de obesidad fue mayor en niños (15,3% frente a 12,7%) y en zonas rurales (17,2% frente a 13,1%), aunque con diferencias estadísticamente no significativas. La obesidad fue más prevalente a los 3 años (16,1%) y menos prevalente a los 6 (11,2%). Tanto en niños como en niñas, la edad (OR=0,30; IC95%: 0,19-0,38 y OR=0,24; IC95%: 0,17-0,36), la altura (OR=1,21; IC95%: 1,16-1,26 y OR=1,25; IC95%: 1,18-1,38) y la zona urbana/rural (OR=0,60; IC95%: 0,34-0,86 y OR=0,48; IC95%: 0,31-0,84) resultaron predictores independientes de obesidad en preescolares de Suzhou, China.

**Conclusiones:**

La tasa de obesidad entre preescolares de Suzhou es del 14,0%. Menor edad, mayor estatura y residir en zona rural fueron identificados como predictors independientes de obesidad en ambos sexos.

## INTRODUCTION

The increasing prevalence of childhood obesity has become a global public health concern^1^. Childhood obesity poses significant risks, including impaired organ function and negative impact on mental and psychological well-being, while increasing the likelihood of adult cardiovascular diseases, such as obesity, hypertension, and hyperlipidemia^2^^-^^4^. Individuals born after the turn of the 21^st^ century are experiencing obesity at younger ages, with greater severity during key developmental stages of childhood^5^. This issue has been further exacerbated by the COVID-19 pandemic, during which lockdowns led to reduce physical activity and increased sedentary behavior among children, contributing to the rise of childhood obesity^6^^,^^7^.

Recent evidence indicates that the majority of overweight in obese children occurs before the age of five and tend to persist into later childhood^8^. Preschoolers who are already overweight are particularly susceptible to becoming obese before adolescence, underscoring the importance of early intervention^9^^,^^10^.

Prevention of obesity is far more effective than treatment, and there is growing global recognition of the need for early prevention efforts^11^. The preschool years are considered a critical window for preventing obesity^12^, as this period is essential for forming core beliefs, behaviors, and healthy habits - such as diet and physical activity - that play a key role in obesity prevention and management^13^. To develop effective prevention strategies, it is crucial to understand the factors influencing during early childhood.

In the United States, childhood obesity rates have tripled over the past decade, with over one quarter of children aged 2-5 years classified as overweight^14^. Similarly, China has seen a marked increase in childhood obesity, particularly in certain urban areas, where prevalence rates have risen by four - to six-fold^15^^-^^17^. In 2020, the prevalence of obesity among children aged 3 to 6 in Suzhou’s Wujiang District was reported at 13.2%. Nonetheless, currently the status of childhood obesity in Suzhou remains limited.

This study aims to assess the current prevalence of obesity among preschoolers in both urban and rural areas of Suzhou, China, and to thoroughly examine the various factors influencing this condition.

## METHODOLOGY

This cross-sectional study assessed the prevalence of obesity among preschool children aged 3 to 6 years in Suzhou, China. Data collection took place between March 1 and May 31, 2023.

Suzhou, located in Jiangsu Province, is a major city recognized for its cultural heritage and rapid economic development. It spans approximately 8,488 square kilometers and has a population exceeding 12 million. The city comprises both urban and rural areas - urban districts feature high population density and developed infrastructure, while rural regions are more agricultural and less densely populated.

The sample size (n=848) was calculated based on the estimated obesity prevalence among preschool children in Suzhou, with a 95% confidence level and a 5% margin of error. Participants were selected using a cluster sampling method from eight kindergartens across Suzhou: two in Gusu District, one in the Industrial Park District, one in town and one village kindergarten in Wujiang District, and three in Zhangjiagang City, one in town and two in villages.

Inclusion criteria were: children aged 3 to 6 years with typical cognitive development and without serious physical illnesses. Exclusion criteria included lack of parental informed consent, with secondary obesity, or incomplete data.

Prior to participation, written informed consent was obtained from parents. An *ad-hoc* questionnaire (Appendix I for English version, Supplementary material for Chinese version) was used to collect information about family background, parental knowledge of obesity, attitudes towards childhood obesity, and obesity-related behaviors in both parents and children. Questionnaires were completed by the children’s parents.

The presence of obesity in children was defined as the dependent variable. All children had their height (in centimeters) and weight (in kilograms) measured. Obesity status (excluding secondary obesity) was determined using Chinese national standards for height and weight assessments in children aged 0-6. Children were classified as: mildly obese if their weight exceeded the reference weight by 20%-30% for their for sex and height, moderately obese if the excess was 30%-50%, and severely obese if their weight was over 50% above the reference value for the same sex and height^18^.

The following were included as independent variables: birth weight (in grams), region (rural/urban), picky eating habits (yes/no), beverage consumption (volume and frequency of different beverages per month), food allergies (yes/no), feeding method (exclusive breastfeeding/mixed feeding/exclusive formula feeding), breastfeeding duration (in months), and physical activity time (total hours per month). Additionally, sex (male/female) and age (in years), were recorded.

### Statistical analysis

Data collected through questionnaires and physical measurements were entered into the Epidate 3.02 database for processing. Quantitative variables are presented as mean and standard deviation (SD), while qualitative as frequencies and percentages. Differences in obesity rates across groups (e.g., by sex, age, and region) were assessed using the Chi-square test. To identify factors associated to obesity, variables with a p-value <0.01 in univariate analysis were entered in a multivariate logistic regression analysis using a stepwise method. Association is presented as odds ratio (OR) and its 95% confidence interval (95%CI). Statistical analyses were performed using the SPSS software version 18.0.

## RESULTS

### Participants

Of the initial 950 preschool children recruited, 102 were excluded due to parental refusal (n=50, 5.3%), incomplete data (n=40, 4.2%), or secondary obesity (n=12, 1.3%). The final sample included 848 children, resulting in a response rate of 89.3%. All participants underwent physical examination and their parents completed the questionnaires.

The sample consisted of 437 boys (51.5%) and 411 girls (48.5%). The distribution by age was as follows: 87 children (10.3%) were 3 years old, 192 (22.6%) were 4 years old, 354 (41.7%) were 5 years old, and 215 (25.4%) were 6 years old.

### Obesity prevalence by sex and age

Among the 848 children, 119 were classified as obese, yielding an overall prevalence of 14.0%, gradually decreasing from mild to severe cases (Table 1). Although the obesity rate was slightly higher among boys compared to girls (15.3% vs. 12.7%), this difference was not statistically significant. Additionally, this distribution of obesity levels did not differ significantly between boys and girls.

**Table 1:** Prevalence of obesity among preschool children, overall and by sex

Gender	n (%)	Obesity rate	Degree of obesity n (%)		
		n (%)	Mild	Moderate	Severe
Boy	437 (51.5)	67 (15.3)	36 (8.2)	17 (3.9)	14 (3.2)
Girl	411 (48.5)	52 (12.7)	28 (6.8)	17 (4.1)	7 (1.7)
Total	848	119 (14.0)	64 (7.5)	34 (4.0)	21 (2.5)
p-value		0.53	0.28	0.75	0.12

Obesity detection rates varied across age groups, with the highest rate observed in 3-year-olds (16.1%) and the lowest in 6-year-olds (11.2%). However, no statistically significant differences were found. Boys exhibited higher prevalence of obesity compared to girls across all four age groups, although these differences were not statistically significant (Table 2).

**Table 2:** Obesity detection rates by age group and sex

Age Group	Global		Boys		Girls		p-value
	n (%)	Obesity Rate	n	Obesity Rate	n	Obesity Rate	
		n (%)		n (%)		n (%)	
3	87 (10.3)	14 (16.1)	40	8 (20.0)	47	6 (12.8)	0.15
4	192 (22.6)	26 (13.5)	93	13 (14.0)	99	13 (13.1)	0.31
5	354 (41.7)	55 (15.5)	187	30 (16.0)	167	25 (15.0)	0.76
6	215 (25.4)	24 (11.2)	117	16 (13.7)	98	8 (8.2)	0.08

### Obesity prevalence in urban and rural areas

Among the 186 rural children, the obesity detection rate was 17.2% (n = 32), compared to 13.1% (n = 87) among the 662 urban children. However, this difference was not statistically significant (p=0.350).

### Factors influencing childhood obesity

In the univariate logistic regression analysis, age, height, birth weight, area of residence, and food allergies were significantly associated with childhood obesity (Table 3).

**Table 3:** Univariate logistic analysis of the influencing factors of childhood obesity

Factors	Boys	Girls	p
	OR (95%CI)	OR (95%CI)	
Age (years)	1.25 (1.08-1.67)	1.30 (1.12-1.72)	0.005
Height (cm)	1.50 (1.03-1.40)	1.55 (1.10-1.60)	0.015
Birth weight (kg)	0.78 (0.64-0.94)	0.82 (0.68-0.98)	0.021
Regions (rural vs urban)	2.00 (1.22-3.30)	1.95 (1.18-3.20)	0.008
Food allergy	1.85 (1.05-3.25)	1.75 (0.98-3.10)	0.045

OR: odds ratio; CI: confidence interval.

In the multivariate analysis, age, height, and area of residence remained significantly associated with obesity after adjusting for other variables (Table 4). Specifically, older age and urban residence were associated with lower odds of obesity, while greater height was associated with higher odds. Children living in urban areas had 40% lower odds of obesity for boys (OR=0.60) and 52% lower odds for girls (OR=0.48) compared to those living in rural areas. Each additional year of age reduced the odds of obesity by 70% in boys (OR=0.30) and 76% in girls (OR=0.24). Each additional centimeter in height increased the odds of obesity by 21% in boys (OR=1.21) and 25% in girls (OR=1.25). Although birth weight and food allergies were significant in the univariate analysis, they were not retained in the final multivariate model due to lack of statistical significance.

**Table 4:** Multivariate logistic regression analysis of factors influencing childhood obesity

Factor	Sex	OR (95%CI)	p-value
Age (years)	Boys	0.30 (0.19-0.38)	<0.01
	Girls	0.24 (0.17-0.36)	<0.01
Height (cm)	Boys	1.21 (1.16-1.26)	<0.01
	Girls	1.25 (1.18-1.38)	<0.01
Regions (Urban vs. Rural)	Boys	0.60 (0.34-0.86)	0.01
	Girls	0.48 (0.31-0.84)	0.02

OR: odds ratio; CI: confidence interval.

## DISCUSSION

This study focused on the prevalence of obesity and its associated factors in kindergarten children (aged 3-6 years) across urban and rural areas of Suzhou City. The results indicate that the obesity rate among preschool children in Suzhou is 14.0%, higher than the 10.4% reported in the *Report on Nutrition and Chronic Diseases of Chinese Residents* (2020). This elevated prevalence highlights the urgent need to address childhood obesity in this region.

The obesity rate is higher in boys than in girls (15.3% vs 12.7%), consistent with findings from previous studies conducted in China and other regions^19^^-^^21^. This gender disparity may be influenced by cultural preferences for a robust physique in boys, often leading to overfeeding and reduced physical activity^22^^-^^24^. Despite the concerning prevalence, most cases were classified as mild obesity, suggesting that early interventions could effectively mitigate this condition. This finding offers optimism that appropriate measures could substantially reduce the health impact of childhood obesity in Suzhou.

Obesity detection rates vary across age groups, with the highest rate among 3-year-olds and the lowest among 6-year-olds. These differences may reflect the gradual increase in both the range and intensity of physical activities as children grow older, supported by studies showing that older children participate in more structured physical activities^12^. This result is confirmed by the decrease in the prevalence of obesity in children between 3 and 6 years. The multivariate logistic model confirmed this trend, showing that with each additional year, the odds of obesity decreases by 73% in boys and 75% in girls.

These results differ from those reported by Ye Hu et al.^25^ in 2022, who found that the prevalence of obesity among children aged 3-6 years increased with age in another Chinese city, Hangzhou. This discrepancy may be attributed to regional variations in lifestyle factors or dietary habits.

As height increased, the risk of obesity rises by approximately 1.21 times in boys (OR=1.21) and 1.25 times in girls (OR=1.25). This positive association may reflect the dual role of growth and appetite in young children, suggesting that those who grow faster and have a stronger appetite may be more susceptible to obesogenic environments^26^. This aligns with previous studies indicating that rapid height growth during early childhood could be a risk factor for obesity, potentially due to increased caloric intake relative to energy expenditure^27^^,^^28^.

Although birth weight showed an association with obesity risk in the univariate analysis, it was not retained in the final multivariate model, as its influence was not statistically significant after adjusting for other variables. This suggests that the association observed initially may have been confounded by other factors. However, the initial finding highlights the importance of birth weight in studies on childhood obesity. Kumar et al.^29^ reported that high birth weight (>3,500 g) increases the risk of childhood obesity, while normal birth weight reduces the risk by up to five times. In our study, birth weight was treated as a continuous variable (measured in grams), differing from studies that categorized it (e.g., high vs. normal). These methodological differences, along with variations in sample characteristics, may partly explain discrepancies across studies. Further research is needed to clarify the role of birth weight in childhood obesity, accounting for potential confounders and methodological variations.

A distinctive aspect of this study is the exploration of the disparities in obesity prevalence and dietary behaviors between urban and rural children. The higher obesity prevalence observed in rural children (17.2%) compared to urban children (13.1%) contrasts with findings from other regions^30^^,^^31^. This disparity may stem from limited access to nutrition education, fewer healthy food options, and reduced availability of structured physical activity programs in rural areas^32^^,^^33^. Nutritional education activities organized in rural schools and communities aim to raise awareness among parents and children about healthy eating habits^34^^,^^35^. Additionally, encouraging schools and communities to host diverse sports activities and competitions can effectively foster children’s interest in physical activity, contributing to the prevention of childhood obesity^36^.

There are several limitations to this study. The use of a cluster sampling method may have introduced selection bias, as the selected kindergartens might not fully capture the socioeconomic diversity of Suzhou. Factors such as family income, parental education level, and regional disparities were not explicitly controlled during the sampling process, potentially limiting the generalizability of the findings. Moreover, some variables were self-reported by parents; for instance, picky eating habits were based on parental responses, which may introduce subjectivity and recall bias. Such biases could distort the relationships between these variables and childhood obesity, thereby affecting the reliability of the conclusions.

In conclusion, this study highlights a concerning obesity prevalence of 14.0% among preschool children aged 3-6 years in Suzhou, China, with higher rates observed among boys and in rural areas. Key factors influencing obesity include age, height, and regional differences, with older children and those living in urban areas showing lower risks of obesity.

These findings underscore the urgent need for targeted interventions, particularly in rural areas, to address dietary habits, physical activity, and nutrition education. Early prevention strategies are critical to mitigate the long-term health impacts of childhood obesity. Future research should further investigate the role of birth weight and other potential risk factors to support the development of more comprehensive obesity prevention programs.

## Figures and Tables

**Figure:**
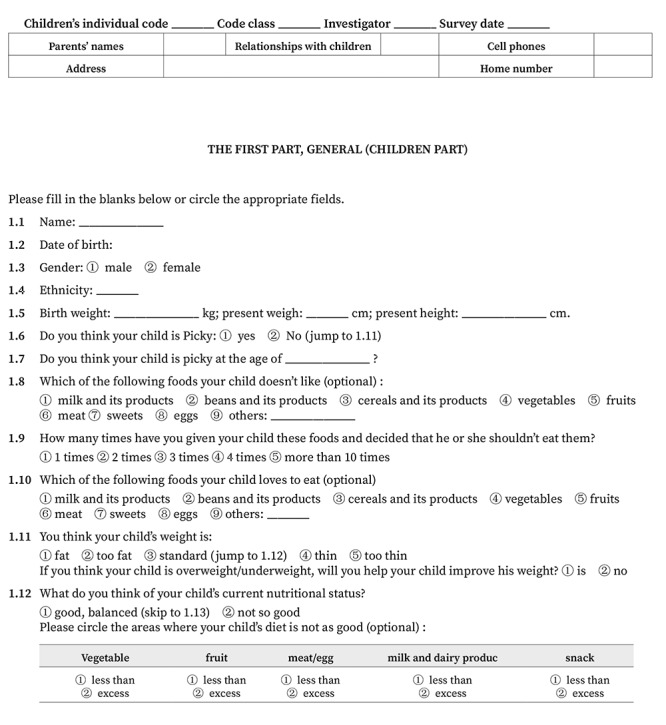

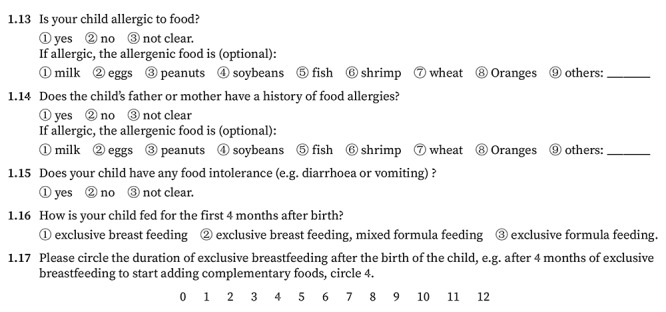

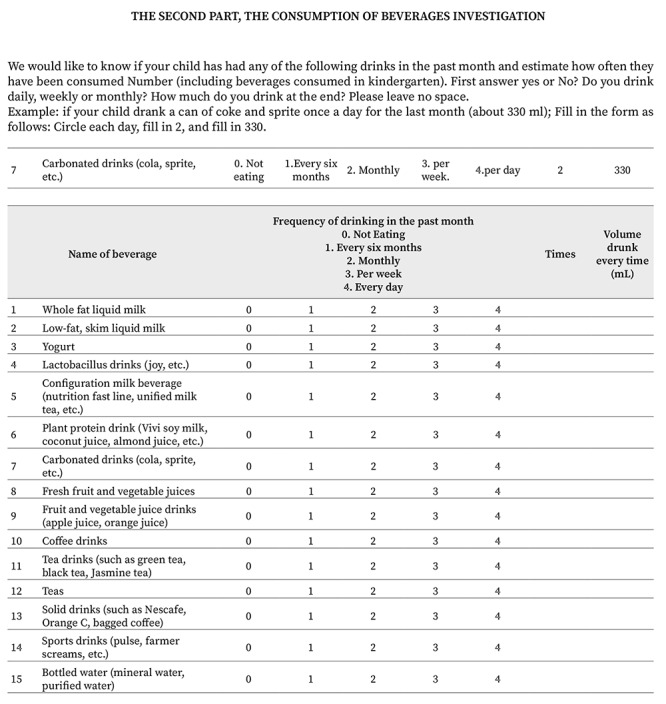

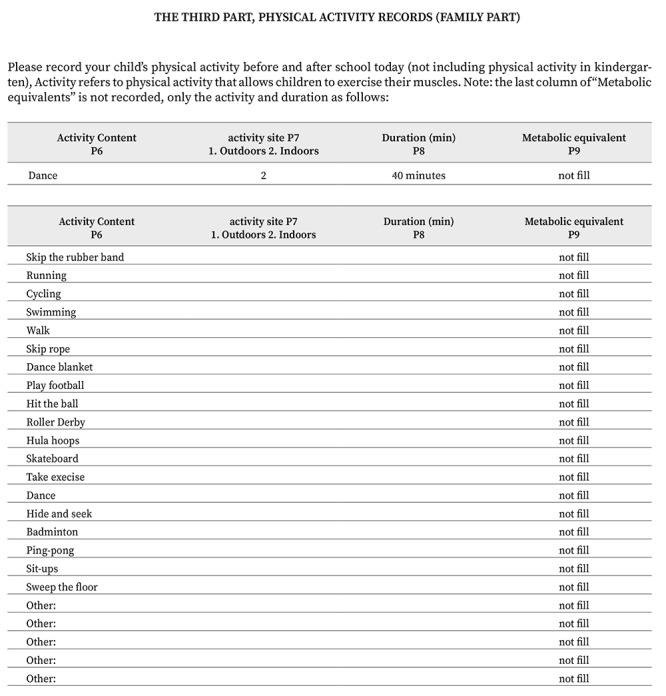

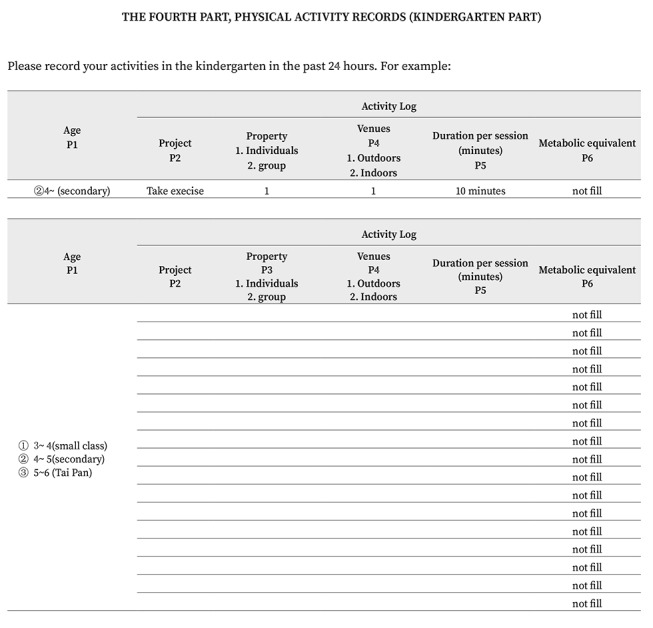
Nutritional status of children aged 3 ~ 6 years in Suzhou (Gusu district, industrial park, Wujiang city, Zhangjiagang city)

## Data Availability

They are available upon request to the corresponding author.
